# Identification of the sex pheromone of *Lutzomyia longipalpis *(Lutz & Neiva, 1912) (Diptera: Psychodidae) from Asunción, Paraguay

**DOI:** 10.1186/1756-3305-2-51

**Published:** 2009-11-02

**Authors:** Reginaldo P Brazil, Norath Natalia Caballero, James Gordon C Hamilton

**Affiliations:** 1 Lab. de Bioquímica e Fisiologia de Insetos - Instituto Oswaldo Cruz, Rio de Janeiro, Brazil; 2 Centre for Applied Entomology and Parasitology, Keele University, Staffordshire, UK

## Abstract

The sand fly *Lutzomyia longipalpis *is the main vector of *Leishmania (L.) infantum *(Nicolle), the causative agent of American visceral leishmaniasis (AVL) in the New World. Male *Lu. longipalpis *have secretory glands which produce sex pheromones in either abdominal tergites 4 or 3 and 4. These glands are sites of sex pheromone production and each pheromone type may represent true sibling species. In Latin America, apart from *Lu. pseudolongipalpis *Arrivillaga and Feliciangeli from Venezuela, populations of *Lu. longipalpis *s.l. can be identified by their male-produced sex pheromones: (S)-9-methylgermacrene-B, 3-methyl-α-himachalene and the two cembrenes, 1 and 2.

In this study, we present the results of a coupled gas chromatography - mass spectrometry analysis of the pheromones of males *Lu. longipalpis *captured in an endemic area of visceral leishmaniasis in Asunción, Paraguay. Our results show that *Lu. longipalpis *from this site produce (S)-9-methylgermacrene-B which has also been found in *Lu. longipalpis *from different areas of Brazil, Colombia and Central America.

## Findings

Visceral leishmaniasis is endemic in several areas of Paraguay with the reports of sporadic cases and consecutive increase in the last few years [1-3]. Asuncion in the Central Department of Paraguay has reported the largest numbers of human cases but other regions more distant from the capital, such as Bella Vista Norte, near the border with Brazil, Encarnacion, near the border with Argentina, and the Departments of Conception and Amambay y San Pedro have been considered as new endemic areas by the Paraguayan Health Secretary [4,5].

As in most endemic areas, *Lutzomyia longipalpis s.l*. is the main vector of *Leishmania (L.) infantum (*Kinetoplastida: Trypanosomatidae), the causative agent of visceral leishmaniasis in South and Central America. Even though *Lu. longipalpis *is recognized as a species complex, no consensus has been established on the number of species present in the New World [6-13].

Field and laboratory observations have shown that, prior to copulation, *Lu. longipalpis s.l*. males wing flutter. This behaviour is associated with pheromone release by males during courtship [14-16]. The sex pheromones are produced in glandular tissue that underlies the cuticle of the abdominal tergites. Those pheromone-disseminating structures are visible, as a pair of pale patches [17,18] on the fourth or third and fourth tergites and have been confirmed as the site of sex pheromone production [19]. There is no relationship between spot morphology and sex pheromone type [20]. Sex pheromones of the *Lu. longipalpis s*pecies complex have been shown to be homosesquiterpenes (C16) or diterpenes (C20) with molecular weights of 218 or 272 respectively. Based on the main terpene component, at present, four different sex pheromone-producing populations (chemotypes) of *Lu. longipalpis *are recognized in Brazil. The homosesquiterpenes have been characterized as 3-methyl-α-himachalene and (*S*)-9-methylgermacrene-B and the diterpene as two cembrene isomers [20-25], These compounds are volatile attractants for conspecific females and may help to maintain species isolation [19,26]. Apart from Brazil and Venezuela, virtually no information has been published on the pheromone types of *Lu. longipalpis *s.l. from different South American countries.

The objective of this study was to collect preliminary information on the sex pheromone of *Lu. longipalpis *from Paraguay.

*Lu. longipalpis were *collected with CDC light traps in a chicken coop over two consecutive nights in an endemic area of VL in Vila Elisa, Asunción (S25°23' 01" W57°36' 60"). After separating males from females and checking species identities by external morphology of their males genitalias, males were placed in glass ampoules prepared from Pasteur pipettes with n-hexane (20 μl) (spectroscopic grade, Sigma Co.) and flame sealed. Prior to analysis, extracts were removed from the Pasteur pipette vials, filtered through glass wool to remove the flies and fly hairs, and the volume reduced under N_2 _to 1 μl. All the chemical analysis was done according to the procedures of Hamilton *et al*. [24]. Fifteen individual males were examined. Mass spectra and gas chromatography retention times were compared with authentic (s) - 9-methylgermacrene-B. Peak enhancement studies were performed by co-injecting extracts of *Lu. longipalpis *from Lapinha (Minas Gerais, Brazil) and *Lu. longipalpis *from Asuncion. GC-MS analysis was carried out on a Hewlett Packard 5890 II+ gas chromatograph with an HP-5MS capillary column, 30 m × 0.25 mm i.d., 0.25 mm film thickness, directly coupled to a Hewlett Packard 5972A bench top mass spectrometer, EI, 70eV, 165°C. Samples were introduced via an on-column injector (40°C). The gas chromatograph (GC) was temperature programmed with an initial 2 min at 40°C, then an increase of 10°C min^-1 ^to a final isothermal period at 250°C (10 min).

To confirm the tentative species identification of male *Lu. longipalpis *made in the field after GC-MS analysis of the hexane extract, all the bodies were preserved in ethanol and were mounted individually on glass slides for detailed morphological examination and species confirmation [27].

This study is the first report of the detailed analysis of the terpene composition of members of the *Lu. longipalpis s.l*. complex in Paraguay and mass spectral data, 218 (M^+^, 22), 165(49), 135(76), 121(100), 119(40), 107(62), 93(71), 91(44), 79(40), 67(78), 41(66), retention time and peak enhancement results showed that Paraguayan *Lu. longipalpis *males produce (s)-9-methylgermacrene-B. This finding confirms the close taxonomic relationship between this population and others found in Brazil largely in the State of Mato Grosso do Sul. Our recent field studies in a VL focus in this state have shown that synthetic (s)-9-methylgermacrene is highly attractive to female *Lu. longipalpis *[28] and this offers the possibility for the development of pheromone based strategies for the control of this vector not only in Brazil but also in Paraguay[29]. Apart from similarity between (s)-9-methylgermacrene-B found in populations from Mato Grosso do Sul and Paraguay, it would be interesting in the future to determine if they share other genetic similarities with the Brazilian 9-methylgermacrene-B populations [30].

## Competing interests

The authors declare that they have no competing interests.

## Authors' contributions

RPB and JGCH conceived the idea. RPB and NNGC collected and identified the sandflies. JGCH analysed the samples. RPB and JGCH wrote the paper.
